# Laceration of Duodenum, with Post Mortem

**Published:** 1877-07

**Authors:** E. V. Stoddard


ART. III.—*Laceration of Duodenum.* By E. V. STODDARD, M.D. At 1 P. M. Oct. 9, I. C. E., æt 33, received a blow upon the Epigastrium from a piece of board about three feet long and six inches wide, thrown back by a circular saw against which it had been placed for the purpose of division. The blow fell in the median line about two inches above the umbilicus. Examination, external, revealed a hardly noticeable abrasion. Nothing else. Patient pale and exsanguine; skin cool; pulse fifty-two and very slow; pain at seat of injury intense; abdominal muscles rigidly contracted; stimulants, and everything taken into the stomach, instantly rejected.

Chloroform, with hot fomentations, and one-sixth grain morphia Sulph., Hypodermically relieved the pain after a short time.

At 6 P. M., easy, p. 84; at 10 P. M., easy, p. 94; at 12 P. M., p. 96; urine by catheter, ℥ NI.

Oct. 10th, 7 A. M.; has been quiet to this time; restless, p. 110; weak, respiration short; constant desire to urinate; no urine by catheter; failed rapidly and died at 9.30 A. M.,—(twenty-one hours after receipt of injury.)

*Post mortem*, nine hours after death; rigor mortis well marked; slight emphysema of subcutaneous cellular tissue of abdomen, chest, neck and face, principally of left side. External marks of injury: a very slight abrasion about two inches above the umbilicus, in the median line; abdomen slightly distended by gas; on opening thorax and abdomen, traces of injury very apparent. The muscles of abdominal wall ecchymotic internally at seat of injury. Small clots of blood lying upon the intestines, below the stomach, and the abdominal cavity contained three pints of bloody fluid and clots. The intestines and omentum highly congested. The stomach appeared normal with the exception of slight congestion of its lower portion. At its pyloric extremity was the seat of lesion; at this point a laceration had occurred, separating the duodenum transversely from the pylorus. The upper margin of the liver showed a slight laceration and contusion. Lungs healthy, except considerable luephysema extending through the mediantinum. The cause of death was evident in the *complete laceration and separation of the duodenum* at the pyloric orifice of the stomach.

